# Kynurenine Pathway Metabolites as Biomarkers for Amyotrophic Lateral Sclerosis

**DOI:** 10.3389/fnins.2019.01013

**Published:** 2019-09-20

**Authors:** Vanessa X. Tan, Gilles J. Guillemin

**Affiliations:** Macquarie University Centre for MND Research, Department of Biological Sciences, Faculty of Medicine and Health Sciences, Macquarie University, Sydney, NSW, Australia

**Keywords:** kynurenine pathway, amyotrophic lateral sclerosis, biomarker development, neurodegeneration, motor neuron disease, neuroinflammation and neurodegeneration, tryptophan

## Abstract

Amyotrophic Lateral Sclerosis (ALS) currently lacks a robust and well-defined biomarker that can 1) assess the progression of the disease, 2) predict and/or delineate the various clinical subtypes, and 3) evaluate or predict a patient’s response to treatments. The kynurenine Pathway (KP) of tryptophan degradation represent a promising candidate as it is involved with several neuropathological features present in ALS including neuroinflammation, excitotoxicity, oxidative stress, immune system activation and dysregulation of energy metabolism. Some of the KP metabolites (KPMs) can cross the blood brain barrier, and many studies have shown their levels are dysregulated in major neurodegenerative diseases including ALS. The KPMs can be easily analyzed in body fluids and tissue and as they are small molecules, and are stable. KPMs have a Janus face action, they can be either or both neurotoxic and/or neuroprotective depending of their levels. This mini review examines and presents evidence supporting the use of KPMs as a relevant set of biomarkers for ALS, and highlights the criteria required to achieve a valid biomarker set for ALS.

## Amyotrophic Lateral Sclerosis

The diagnosis of amyotrophic lateral sclerosis (ALS) can only be fully confirmed by the post mortem detection of ALS-associated protein inclusions such as TDP-43 and SOD1 (Turner et al., 2013). Coupled with the spectrum of symptoms seen in the clinical presentation of ALS, the diagnosis of ALS relies on presentation to a neurologist and the elimination of other neurological and/or muscular diseases such as Kennedy’s disease or myasthenia gravis, based on the El Escorial criteria that requires the assessment of disease progression (Brooks et al., 2000; Lambrechts et al., 2007; Al-Chalabi et al., 2016; Hardiman et al., 2017). This results in the average time from onset of symptoms after diagnosis of ALS being 10 months, in a disease with survival of 24–48 months (Chiò et al., 2009; Hardiman et al., 2017).

Defined as characteristic that is objectively measured and evaluated as an indicator of normal biological process, pathogenic process, or a pharmacogenomic process to therapeutic intervention, biomarkers include genomics, proteomics, metabolomics, neurophysiology, and neuroimaging (Ganesalingam and Bowser, 2010; Turner et al., 2011). The lack of a reliable biomarker for ALS hampers a rapid, definitive diagnosis of disease, determination of ALS subtypes, monitoring of disease progression in patients, and limits the ability of clinicians and scientists to achieve an unbiased assessment of the efficiency of new treatments (Turner et al., 2009; Ganesalingam and Bowser, 2010). For patients and their families, a sensitive and specific biomarkers could allow detection of ALS at early stages, and allow the prognosis of the clinical subtype of ALS to predict disease aggressivity and subtype (Ganesalingam and Bowser, 2010; Al-Chalabi et al., 2016). This research gap in biomarker discovery and development for ALS comes not only as an impediment for patients and their families, but also at a cost to the pharmaceutical industries, through the monitoring of drug effects and disease progression in clinical trials. In particular, the repeated failure of drugs demonstrating clinical efficacy, and the inability to detect improvements, or non-improvements rapidly (Aggarwal and Cudkowicz, 2008; Ganesalingam and Bowser, 2010; Petrov et al., 2017).

## The Kynurenine Pathway

**FIGURE 1:**
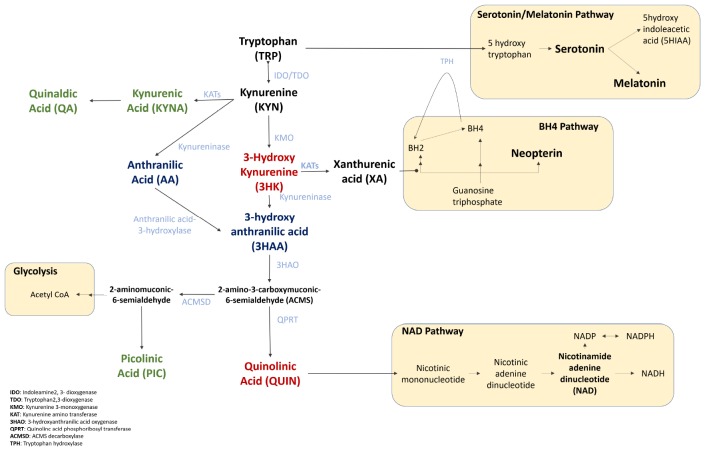
The Kynurenine Pathway and its downstream pathways of Serotonin/Melatonin, BH4, Glycolysis, and NAD+. Tryptophan is converted into serotonin and melatonin, that regulate mood and sleep. The tetrahydrobiopterin (BH4) pathway interacts with the KP in three ways, (1) the sharing of the enzyme TPH that degrades tryptophan, (2) the inhibition of a key BH4 pathway enzyme, sepiapterin reductase, (3) both BH4 and KP are induced by inflammatory cytokines. Tryptophan also feeds into the glycolysis cycle via ACMS, affecting ATP production. Finally, the KP is the *de novo* synthesis pathway of NAD+ which is associated with cellular energy, repair and fatigue. The key KPMs are bolded, neurotoxic metabolites represented in red, neuroprotective metabolites in green, and dual functioning in blue.

One of the hallmarks of ALS is the presence of neuroinflammation and the kynurenine pathway (KP) is known to be strongly induced by inflammatory cytokines such as IFN-γ (McGeer and McGeer, 2002; Moffett and Namboodiri, 2003; Chen et al., 2010; Oxenkrug, 2011). The KP is the major route of tryptophan (TRP) catabolism, and feeds into the serotonin pathway, immune related tetrahydrobiopterin (BH4) pathway, glycolysis, and *de novo* nicotinamide adenine dinucleotide (NAD+) pathway (Figure 1) (Stone, 1993; Grant et al., 2010; Oxenkrug, 2013; Sasaki, 2019); linking it to fatigue, depression, inflammation, and decrease in energy metabolism (Sandyk, 2006; Grant et al., 2010; Oxenkrug, 2013).

The essential amino acid tryptophan originates from the diet, if which up to 85% is bound to albumin in blood circulation, and 99% metabolized in the liver (Quagliariello et al., 1964; Yuwiler et al., 1977; Badawy, 2017). Activation of the KP is achieved by the triggering of the first enzyme of the pathway, indoleamine 2,3 dioxygenase (IDO1) (Guillemin et al., 2005c; Badawy, 2017). This results in the production of several neuroactive metabolites such as the excitotoxins quinolinic acid (QUIN), and 3-hydroxykynurenine (3HK) by activated monocytic cells (Guillemin et al., 2003b); and the neuroprotective kynurenic acid (KA) and picolinic acid (PIC) by astrocytes and neurons, respectively (Heyes et al., 1988; Beninger et al., 1994; Guillemin et al., 2001, 2007; Badawy, 2017). The KP is active in most cell types, particularly in the liver (Takikawa et al., 1986; Heyes et al., 1997), and is highly activated in monocytic cells during inflammation (Jones et al., 2015). Only a limited number of KP can cross the blood brain barrier (BBB). TRP, Kynurenine (KYN), 3HK, anthranilic acid (AA) are actively transported by the large neutral amino acid carrier system; and others via passive diffusion (Fukui et al., 1991; Ruddick et al., 2006). This indicates that peripheral activation of the KP by inflammation can be translocated to the central nervous system (CNS), altering immune regulation and increasing neurotoxicity (Owe-Young et al., 2008). In the CNS, most cells contain the complete set of KP enzymes, and are capable of degrading TRP (Guillemin et al., 2005c; Lee et al., 2017). However, neurons, astrocytes and oligodendrocytes are incapable of synthesizing QUIN, only activated microglia and infiltrating macrophages produce QUIN (Guillemin et al., 2000; Lim et al., 2007).

The concept of using kynurenine pathway metabolites (KPMs) as markers for diseases dates back to the 1950s (Musajo et al., 1955; Tompsett, 1959), where excretion of KPMs were observed in the urine of patients diagnosed with cancer, rheumatoid arthritis, cardiovascular events and fevers (Musajo et al., 1955; Takahashi et al., 1956; Tompsett, 1959; McMillan, 1960; McManus and Jackson, 1968; Mawatari et al., 1995). More recently, the KP is investigated mostly in other liquid biopsies such as serum and plasma (Lewitt et al., 2013). The levels of the KPMs has been shown to be well correlated between the cerebrospinal fluid (CSF) and blood (Curzon, 1979; Chen et al., 2010; Myint, 2012; Jacobs et al., 2019), however, they are not always identical; and only few studies (Curzon, 1979; Widner et al., 2002; Chen et al., 2010; Zuo et al., 2016; Havelund et al., 2017; Lim et al., 2017; Jacobs et al., 2019) correlate the KP levels in different biofluids from the same patients at the same time. KPMs have been historically measured using thin layer chromatography, and detected under UV light, or via radioactive metabolites (Musajo et al., 1955, 1956; McMillan, 1960; McManus and Jackson, 1968; Shibata, 1988). Today, KPMs are more often measured using more sensitive methods and equipment such as high performance liquid chromatography (HPLC), Gas chromatography mass spectrometry (GCMS), and liquid chromatography tandem mass spectrometry (LC-MS/MS) (Heyes and Markey, 1988; Bizzarri et al., 1990; Smythe et al., 2003; de Jong et al., 2009; Pedersen et al., 2013; Miller et al., 2018). The most commonly measured KPMs are TRP, KYN, and KYNA, and are often presented as ratios. As they are small molecules, the KPMs such as KYN, KYNA, Xanthurenic acid (XA) and AA have been shown to be stable. With the exception of 3-hydroxy anthranilic acid (3HAA), which is known to be particularly unstable over time and sensitive to light (Darlington et al., 2010; Midttun et al., 2014).

*Ex vivo*, the KPMs have been measured using immunohistochemistry (IHC) in tissue sections (Guillemin et al., 2005a; Steiner et al., 2011; Lim et al., 2013). More recently, techniques such as tissue-based Matrix-assisted laser desorption/ionization (MALDI) Mass spectrometry Imaging (MSI) and tissue microarray has been used to not only detect, but localize the presence of TRP and KYN in tumors *ex vivo* (Puccetti et al., 2015; Ait-Belkacem et al., 2017). This specific localization will allow for focal observation of KPMs changes within tissue, and targeted applications of monitoring and altering of the KP if this can be translated *in vivo*.

Using the levels of KPMs, the activity of their associated enzymes can be derived as a proxy of the concentrations of direct derivatives of the metabolites as a ratio (Darlington et al., 2007; Sathyasaikumar et al., 2011; Lim et al., 2013) – most commonly measuring IDO1 via the Kynurenine: Tryptophan ratio (K/T ratio; KTR); or via direct enzymatic assays (Sathyasaikumar et al., 2011). Although few studies have looked at the direct correlation between metabolite ratio and enzymatic concentrations (Baran et al., 1999).

*In vitro*, neurotoxic KPMs such as QUIN and 3HK, have been shown to induce neurodegeneration and neuronal cell death through excitotoxicity, *N*-methyl-D-aspartate (NMDA) receptor antagonism, increased glutamate release, and the production of reactive oxygen species (Kim and Choi, 1987; Koh and Choi, 1988; Khaspekov et al., 1989; Nakagami et al., 1996; Shoki et al., 1998; Guidetti and Schwarcz, 1999; Leipnitz et al., 2007; Guillemin, 2012b; Kalonia et al., 2012; Pierozan et al., 2015). The neurotoxic mechanisms of QUIN is well established, and overlaps with mechanisms of neurodegeneration in ALS such as excitotoxicity, hyperphosphorylation, and protein dysfunction (Pierozan et al., 2010; Guillemin, 2012a; Lee et al., 2017). Some of the KPMs such as KYNA, PIC, and 3HAA have neuroprotective and immunomodulatory properties (Foster et al., 1984; Behan and Stone, 2000; Grant et al., 2009; Krause et al., 2011; Lugo-Huitrón et al., 2011). Other KPMs such as 3HAA, have both neurotoxic and neuroprotective functions depending on their relative concentrations (Colín-González et al., 2013; Pérez-González et al., 2017). The KPMs can influence each other levels (Perkins and Stone, 1982; Jhamandas et al., 1990), and the balance of KPMs is crucial for managing the equilibrium between neurotoxicity and neuroprotection. The dysregulation of KPMs, especially excessive QUIN production, has been correlated with variations of other neuroinflammatory markers (Heyes et al., 1992; Guillemin et al., 2003a; Kalonia et al., 2011), making the modulation of KPMs a plausible target for the regulation of the immune response within the CNS (Stone et al., 2012; Bohár et al., 2015; Jacobs and Lovejoy, 2018).

Using these modern techniques, the KP has been investigated as a marker for progression, severity, and prognostic for diseases such as systemic lupus erythematosus (Perl, 2015; Åkesson et al., 2018), cancers (Jin et al., 2015; Zuo et al., 2016; Xie et al., 2017; Huang et al., 2018; Liu et al., 2018; Khan et al., 2019), cardiovascular disease (Sun et al., 2013; Zuo et al., 2016), lung cancer and chronic obstructive pulmonary disease (Chuang et al., 2014; Zinellu et al., 2018), chronic kidney disease and diabetes (Hirayama et al., 2012; Zhao, 2013), acquired immunodeficiency syndrome (AIDS) and HIV-dementia (Fuchs et al., 1990; Heyes et al., 1991; Sardar et al., 2002; Guillemin et al., 2005b; Favre et al., 2010; Lee et al., 2016; Wang et al., 2019), pancreatic cysts (Park et al., 2013), acute myeloid leukemia and lymphomas (Giusti et al., 1996; Finger et al., 2017), vitamin levels (Midttun et al., 2014), tuberculosis (Weiner et al., 2012; Feng et al., 2015), malaria (Medana et al., 2003), irritable bowel syndrome (IBS) (Clarke et al., 2012; Gupta et al., 2012), rheumatoid arthritis (Spiera and Vallarino, 1969; Schroecksnadel et al., 2003), growth deficits (Kosek et al., 2016), obesity (Mangge et al., 2014), and preeclampsia (Nilsen et al., 2012). In the nervous system, the KP has been shown to associate with pathologies such as stroke (Darlington et al., 2007), schizophrenia (Müller and Schwarz, 2006; Kegel et al., 2014; Oxenkrug et al., 2016), Parkinson’s (Ogawa et al., 1992; Widner et al., 2002; Lewitt et al., 2013; Havelund et al., 2017), neuropsychiatric disorders such as depression and stress (Mackay et al., 2009; Gabbay et al., 2010; Olsson et al., 2010; Steiner et al., 2011; Kocki et al., 2012; Erhardt et al., 2013; Comai et al., 2016; Küster et al., 2017; Huang et al., 2018; Kuwano et al., 2018), suicide (Erhardt et al., 2013; Bay-Richter et al., 2015; Brundin et al., 2016), multiple sclerosis (Rejdak et al., 2002; Lim et al., 2017), Alzheimer’s disease (Guillemin et al., 2005a; Hartai et al., 2007), Huntington’s disease (Schwarcz et al., 1988; Beal et al., 1990; Stoy et al., 2005; Byrne and Wild, 2016), brain tumors (Adams et al., 2012, 2014), Autism Spectrum Disorders, and Attention Deficit Hyperactivity Disorder (ADHD) (Aarsland et al., 2015; Bryn et al., 2017). More recently, studies have demonstrated that the KPMs could be used for the prognosis of MS, and also to differentiate between disease subtypes (Aeinehband et al., 2015; Lim et al., 2017).

## Amyotrophic Lateral Sclerosis and Kynurenine-Associated Pathways

The levels of KPMs are known to be dysregulated in the serum, CSF, and tissue of ALS patients (Ilzecka et al., 2003; Chen et al., 2010) (Table 1). The first study by Ilzecka et al. (2003) investigated the presence of KYNA in ALS patients and matching healthy controls. Broadly, the results did not show any significant differences in the levels of KYNA between patients and controls in either serum or CSF. However, CSF KYNA was higher in (1) patients with severe clinical status; and (2) in patients with bulbar onset, compared to patients with limb onset. The authors concluded that this increase likely associated with the neuroprotective role of KYNA. The authors also showed that the concentrations of KYNA in CSF and in serum were not correlated, indicating that KYNA in the CNS is mostly produced in the brain by astrocytes (Guillemin et al., 2001), and this confirms that KYNA is able to cross the BBB and may be imported from the PNS. This is supported by the presence of astrogliosis as part of the neuroinflammatory features found in ALS brain. In 2010, our team reported increased levels of TRP, KYN, and QUIN in both CSF and serum. This study did not investigate KYNA or astrogliosis as Ilzecka et al. did, however, does confirm the neuroinflammatory status in ALS patients, with presence of activated microglia and activation of the KP in the motor cortex.

**TABLE 1:** Summary of Kynurenine Pathway metabolite levels in controls and ALS patients collated from Ilzecka and Chen.

**References**	**KPM**	**Population**	**Serum**	**CSF**	**Trend observed**	
					**Serum**	**CSF**
Ilzecka	Kyna pmol/ml	Control (*n* = 14)	59.6 ± 20.5	2.41 ± 1.7		
		ALS (*n* = 16)	57.8 ± 35.0	1.59 ± 0.9	Mild > Severe	Control < Bulbar
		Bulbar (*n* = 6)	59.5 ± 39.3	3.61 ± 2.0	Control > Severe	Bulbar > Limb
		Limb (*n* = 10)	59.6 ± 31.2	1.70 ± 1.0	clinical status	Control < Severe
		Mild clinical status (*n* = 6)	81.6 ± 41.2	1.75 ± 09		clinical status
		Severe clinical status (*n* = 8)	39.9 ± 14.7	3.26 ± 2.1		
Chen	TRP (μM)	Control (*n* = 17)	75.0 ± 10.5	2.58 ± 0.16	Control < ALS^∗∗^	Control < ALS^∗∗^
		ALS(140)	143.3 + 5.6	5.0 ± 0.2		
		sALS (*n* = 133)	133.3 ± 6.0	4.67 ± 0.19		
		fALS (*n* = 7)	166.4 ± 20.7	5.20 ± 0.87		
		Bulbar (*n* = 31)	128.2 ± 10.6	4.58 ± 0.33		
		Limb (*n* = 109)	137.3 ± 6.9	4.73 ± 0.22		
	KYN (μM)	Control (*n* = 17)	2.52 ± 0.19	0.027 ± 0.00	Control < ALS^∗∗^	Control < ALS^∗∗^
		ALS(140)	4.0 + 0.2	0.23 + 0.02		
		sALS (*n* = 133)	4.05 ± 0.21	0.22 ± 0.01		
		fALS (*n* = 7)	3.24 ± 0.36	0.26 ± 0.05		
		Bulbar (*n* = 31)	3.99 ± 0.29	0.22 ± 0.02		
		Limb (*n* = 109)	4.00 ± 0.24	0.21 ± 0.03		
	PIC (μM)	Control (*n* = 35)	2.4 ± 0.4	0.51 ± 0.11	Control > ALS^∗^	Control > ALS (*p* = 0.09)
		ALS(140)	1.4 + 0.1	0.36 + 0.03		
		sALS (*n* = 133)	1.46 ± 0.13	0.35 ± 0.07		
		fALS (*n* = 7)	1.80 ± 0.51	0.60 ± 0.21		
		Bulbar (*n* = 31)	1.45 ± 0.16	0.30 ± 0.06		
		Limb (*n* = 109)	1.49 ± 0.10	0.35 ± 0.07		
	QUIN (μM)	Control (*n* = 35)	0.30 ± 0.03	0.038 ± 0.004	Control < ALS^∗^	Control < ALS^∗^
		ALS(140)	0.37 + 0.02	0.053 + 0.005		
		sALS (*n* = 133)	0.38 ± 0.02	0.05 ± 0.01		
		fALS (*n* = 7)	0.36 ± 0.04	0.04 ± 0.01		
		Bulbar (*n* = 31)	0.43 ± 0.04	0.04 ± 0.01		
		Limb (*n* = 109)	0.36 ± 0.02	0.05 ± 0.01		
	IDO Activity (K/T ratio)	Control (*n* = 17)	0.039 ± 0.004	0.011 ± 0.001		Control < ALS
		ALS (*n* = 40)	0.037 ± 0.0025	0.044 + 0.002		
		sALS (*n* = 133)	0.04 ± 0.00	0.04 ± 0.00		
		fALS (*n* = 7)	0.02 ± 0.00	0.04 ± 0.01		
		Bulbar (*n* = 31)	0.04 ± 0.00	0.04 ± 0.00		
		Limb (*n* = 109)	0.04 ± 0.00	0.04 ± 0.00		

*Mild clinical status defined as mild to moderate according to Munsat, Severe clinical status defined as severe to terminal according to Munsat. KMP, KP metabolites; Kyna, kynurenic acid; TRP, tryptophan; KYN, kynurenine; PIC, picolinic acid; QUIN, quinolinic acid; IDO, indoleamine dioxygenase; K/T ratio, kynurenine/tryptophan ratio; sALS, sporadic ALS; fALS, familial ALS. ^∗^*p* < 0.05; ^∗∗^*p* < 0.0001.*

Other studies have indirectly reported associations between ALS and the KPMs. Jhamandas et al. (1990) showed that injections of the excitotoxin QUIN and 3HAA, directly into the rat brain, triggers a decrease in choline acetyltransferase (ChAT) activity, and that KYNA, PIC, quinaldic acid (QUINA), and AA co-injections could antagonize the QUIN-induced neurotoxicity. In addition, QUIN injections were associated with neuronal loss, but also glial proliferation, highlighting the important roles played by KPMs in neuroinflammation and glial activation in ALS.

Aside from the KP, tryptophan is also metabolized by pinealocytes into serotonin (5-HT), and then melatonin, a serotonin downstream metabolite. Pinealocytes are external to the BBB, and thus directly affected by the KP in the periphery, but not directly by the KP in the CNS (Ruddick et al., 2006). Within the brain, serotonin is modulated by tryptophan levels. A decrease in serotonin levels has been linked to depression through tryptophan depletion (Owens and Nemeroff, 1994; Ruddick et al., 2006; Maes et al., 2011), and with the decrease of melatonin, and sleep disturbances, which are both symptoms in ALS patients (Sandyk, 2006). Furthermore, motoneurons affected in ALS are heavily innervated by serotoninergic neurons; whereas those resistant to ALS-associated degeneration are less innervated by serotonin neurons, possibly linking serotonin with induction of neuronal excitability and neurodegeneration. The roles of serotonin in ALS has been reviewed (Sandyk, 2006). Melatonin has been shown to confer neuroprotection in ALS patients and Cu/Zn superoxide dismutase (SOD1) mice models, likely by decreasing systemic oxidative stress, caspase activation, and by increasing ATP availability to increase cell repair mechanisms to limit neuronal death (Weishaupt et al., 2006; Zhang et al., 2013).

The metabolic pathway of tryptophan degradation also feeds into the cell’s energy metabolism through the production of NAD+ and glycolysis. Its dysregulation increases the risk for the development of neurodegenerative diseases as many repair and neuroprotective systems perform at a suboptimal level. NAD+ depletion can lead to fatigue (Procaccini et al., 2016; Camandola and Mattson, 2017; Sasaki, 2019). Altered energy metabolism has also been investigated in ALS (Dupuis et al., 2004; Ngo and Steyn, 2015), and has been shown to be altered by QUIN via the respiratory chain and Krebs cycle (Ribeiro et al., 2006; Colín-González et al., 2015). The NAD+ pathway represents an important therapeutic avenue, and is being targeted using precursors such as nicotinamide phosphoribosyl transferase, or nicotinamide ribosyl directed at ageing, neurodegeneration, and in particular, axonal degeneration (Sasaki et al., 2006; Imai and Yoshino, 2013; Verdin, 2015; Pehar et al., 2018).

A pathway that has been understudied in ALS is the tetrahydrobiopterin (BH4) pathway (Figure 1). Interconnected to the KP via the modulatory effect of XA, and as a co-substrate for tryptophan hydroxylase (Zhang et al., 2006; Cronin et al., 2018), studies on BH4 have largely focused on inflammation, pain and neuroprotection (Oxenkrug, 2007; Ghisoni et al., 2015; Cronin et al., 2018). BH4 is strongly associated with neuroinflammation, and is also an essential co-factor in nitric oxide synthases in oxidative stress (Sakai et al., 1995; Guix et al., 2005; Cronin et al., 2018) - both pathological features present in ALS. Several reports have associated BH4 with neurodegeneration, such as the differential methylation of BH4 in monozygotic twins discordant for ALS (Young et al., 2017); and particularly in Parkinson’s Disease (Choi et al., 2004; Foxton et al., 2007; Yoon et al., 2010).

With all these evidences associating the KP in ALS, especially the unbalance between neuroprotective and neurotoxic metabolites, the KPMs represent a relevant set of biomarkers to characterize disease subtypes and to assess disease progression. As mentioned previously, such biomarkers are lacking especially for the response to treatments and for testing new drugs in clinical trials. One of the main reasons supporting the role of KPMs as a biomarker for ALS is its association with neuroinflammation. The KTR (indication of IDO activity, and thus KP activation) is a very sensitive and specific marker for inflammation. This KTR ratio is well suited as a surrogate progressive, or end-point marker for neuroinflammation. Apart from CSF, body fluids such as blood and urine are easiest to collect. Measurement of KPMs levels in blood present a rapid and reliable set of markers as there are validated quantification methods, and they are stable. However, there are still some limitations using the KPMs as a biomarker for diseases.

Firstly, a potential pitfall using the KPMs as a biomarker for neurological diseases and psychological disorders is that KP activation is not specific of one disease as it is present in all neuroinflammatory diseases. Thus, the KP cannot be used a diagnostic marker, but is relevant as a prognostic/progression marker, and to identify disease subtypes. Diagnostically, the KPMs still have a great potential as a confirmatory biomarker in conjunction with a shortlisted clinical diagnosis, or subtype. For example in MS, we were able to differentiate MS subtypes from patients diagnosed with MS (Lim et al., 2017). Similarly, when a patient is suspected to have ALS, or has been diagnosed with ALS by a neurologist, the KPMs can be used to differentiate between disease subtypes (e.g., bulbar or lower motor neuron symptoms) and be able to differentiate between patients predicted to be fast or slow progressors. The addition of other inflammatory markers such as cytokines, chemokines, C Protein Reactive, etc, in combination with KPMs would increase the sensitivity and specificity of the biomarker set.

Secondly, the biological functions of all the KPMs are not fully understood – it is a very complex system that is intertwined other regulatory pathways such as BH4 (Cronin et al., 2018), and ultimately regulate the immune system. Further, there is only a limited direct correlation between enzymatic activities and the metabolite formation and their ratios. This is not a key issue in using the KP as surrogate biomarkers, as the crux is that the KPM ratios (ratios of the bioactive metabolites) are what confer biological activity and biomarker association; rather than the function of measuring the enzyme activity. The levels of KPMs in the general population has been directly investigated by Zuo et al. (2016) (*n* = 7015) and Gostner et al. (2015) (*n* = 100), which showed that some KPMs are influenced by both age (KTR, KYN, HAA), and gender (TRP) (de Bie et al., 2016a, b). Further, tryptophan has been shown to increase through to adolescence (Lepage et al., 1997) and in adulthood (Mangge et al., 2014). An earlier study by Medana et al. (2003) investigated the KPMs in Malawian children and Vietnamese adults who were affected by Malaria, showing that increases in QA and PIC in both populations could predict a fatal outcome. On the contrary, differences in KA levels in Malawian children as compared to Vietnamese adults (Medana et al., 2003), although it is unclear if this difference was attribute to age, disease, or ethnicity. Further, the correlation of the KPMs in different biofluids need to be better established for correlation and pathway studies. Urine represents the ideal biofluid as it is non-invasive. However, it is not homeostatic, and apart from early studies when the KP was discovered in urine (Musajo et al., 1955, 1956; Tompsett, 1959; McMillan, 1960; Mawatari et al., 1995), only few recent studies have analyzed the KPMs in urine (Fukuwatari et al., 2004; Pedersen et al., 2013; Dolina et al., 2014). Recent research mostly use serum or plasma to assess the KPMs (Darlington et al., 2007; Favre et al., 2010; Hirayama et al., 2012; Aarsland et al., 2015; Comai et al., 2016; Oxenkrug et al., 2016; Lim et al., 2017), and some when available, CSF (Erhardt et al., 2013; Havelund et al., 2017; Sühs et al., 2019). Further, the KP has been proposed as therapeutic intervention for neurodegenerative diseases such as ALS, and has been well reviewed (Füvesi et al., 2012).

As for other potential candidate biomarkers for ALS, the need for defined classifications of ALS subtypes or stages of disease progression (Gil et al., 2017) is critical. Standardized operation procedures for a defined analysis of progression rate, imaging, biopsy retrieval and storage, and biomarker analysis techniques need to be implemented to ensure consistency across centers to achieve an objective assessment. Biobanks storing clinical and biopsies of patient and control samples will be crucial to achieving the aim of a clinically applicable biomarker for ALS.

## Conclusion

Overall, the KPMs have potential to be used as a sensitive and specific biomarker for patients diagnosed with ALS. Such markers would also have the ability to be used for surrogate clinical and prognostic biomarkers as we previously demonstrated for MS (Lim et al., 2017) and Alzheimer’s disease (Chatterjee et al., 2018; Jacobs et al., 2019). The strong correlation of the KP with neuroinflammation, depression, and immune regulation makes it a valid candidate as a surrogate biomarker for ALS, for disease progression (fast/slow progressors) and possibly disease subtyping. Combining the KPM levels together with (1) other markers of inflammation or neurodegeneration, (2) clinical information, and (3) imaging would strongly increase both sensitivity and specificity of the biomarker set.

## Author Contributions

VT wrote the first draft of the manuscript. Both authors contributed to manuscript revision and read and approved the submitted version.

## Conflict of Interest

The authors declare that the research was conducted in the absence of any commercial or financial relationships that could be construed as a potential conflict of interest.

## Abbreviations

3HAA: 3-hydroxyanthranilic acid
3HK: 3-hydroxykynurenine
5-HT: serotonin
AA: anthranilic acid
ALS: amyotrophic lateral sclerosis
BBB: blood brain barrier
BH4: tetrahydrobiopterin
ChAT: choline acetyltransferase
CNS: central nervous system
CSF: cerebrospinal fluid
GCMS: gas chromatography mass spectrometry
HPLC: high performance liquid chromatography
IHC: immunohistochemistry
KP: kynurenine pathway
KPM: KP metabolites
KTR: kynurenine: tryptophan Ratio
KYN: kynurenine
KYNA: kynurenic acid
LC-MS/MS: liquid chromatography tandem mass spectrometry
MS: multiple sclerosis
NAD+: nicotinamide adenine dinucleotide
NMDA: *N*-methyl -D-aspartate
PIC: picolinic acid
QUIN: quinolinic acid
QUINA: quinaldic acid
SOD1: Cu/Zn superoxide dismutase
TRP: tryptophan
XA: xanthurenic acid.
